# QuasiFlow: a Nextflow pipeline for analysis of NGS-based HIV-1 drug resistance data

**DOI:** 10.1093/bioadv/vbac089

**Published:** 2022-11-28

**Authors:** Alfred Ssekagiri, Daudi Jjingo, Ibra Lujumba, Nicholas Bbosa, Daniel L Bugembe, David P Kateete, I King Jordan, Pontiano Kaleebu, Deogratius Ssemwanga

**Affiliations:** Department of General Virology, Uganda Virus Research Institute, Entebbe 31405, Uganda; Department of Immunology and Molecular Biology, Makerere University, Kampala 10206, Uganda; Department of Computer Science, Makerere University, Kampala 10207, Uganda; African Center of Excellence in Bioinformatics and Data Intensive Sciences, Makerere University, Kampala 10207, Uganda; Department of Immunology and Molecular Biology, Makerere University, Kampala 10206, Uganda; Medical Research Council (MRC)/Uganda Virus Research Institute (UVRI) and London School of Hygiene and Tropical Medicine (LSHTM) Uganda Research Unit, Entebbe 31405, Uganda; Medical Research Council (MRC)/Uganda Virus Research Institute (UVRI) and London School of Hygiene and Tropical Medicine (LSHTM) Uganda Research Unit, Entebbe 31405, Uganda; Department of Immunology and Molecular Biology, Makerere University, Kampala 10206, Uganda; School of Biological Sciences, Georgia Institute of Technology, Atlanta, GA 30332, USA; Department of General Virology, Uganda Virus Research Institute, Entebbe 31405, Uganda; Medical Research Council (MRC)/Uganda Virus Research Institute (UVRI) and London School of Hygiene and Tropical Medicine (LSHTM) Uganda Research Unit, Entebbe 31405, Uganda; Department of General Virology, Uganda Virus Research Institute, Entebbe 31405, Uganda; Medical Research Council (MRC)/Uganda Virus Research Institute (UVRI) and London School of Hygiene and Tropical Medicine (LSHTM) Uganda Research Unit, Entebbe 31405, Uganda

## Abstract

**Summary:**

Next-generation sequencing (NGS) enables reliable detection of resistance mutations in minority variants of human immunodeficiency virus type 1 (HIV-1). There is paucity of evidence for the association of minority resistance to treatment failure, and this requires evaluation. However, the tools for analyzing HIV-1 drug resistance (HIVDR) testing data are mostly web-based which requires uploading data to webservers. This is a challenge for laboratories with internet connectivity issues and instances with restricted data transfer across networks. We present QuasiFlow, a pipeline for reproducible analysis of NGS-based HIVDR testing data across different computing environments. Since QuasiFlow entirely depends on command-line tools and a local copy of the reference database, it eliminates challenges associated with uploading HIV-1 NGS data onto webservers. The pipeline takes raw sequence reads in FASTQ format as input and generates a user-friendly report in PDF/HTML format. The drug resistance scores obtained using QuasiFlow were 100% and 99.12% identical to those obtained using web-based HIVdb program and HyDRA web respectively at a mutation detection threshold of 20%.

**Availability and implementation:**

QuasiFlow and corresponding documentation are publicly available at https://github.com/AlfredUg/QuasiFlow. The pipeline is implemented in Nextflow and requires regular updating of the Stanford HIV drug resistance interpretation algorithm.

**Supplementary information:**

Supplementary data are available at *Bioinformatics Advances* online.

## 1 Introduction

Next-generation sequencing (NGS) is becoming more popular than Sanger sequencing for HIV-1 drug resistance (HIVDR) genotypic testing mainly due to its ability to identify low abundance variants (Manyana *et al.*, 2021). These variants exist in <20% of the entire HIV-1 viral population and are commonly referred to as HIV-1 minority variants (Li, 2014). Drug resistance mutations in minority HIV-1 variants have been associated with treatment failure across antiretroviral drug classes, mostly especially for non-nucleoside reverse transcriptase-based regimens (Raymond *et al.*, 2018). Therefore, NGS-based HIVDR testing has great potential in uncovering novel insights of clinical significance for HIV-1 patients on antiretroviral treatment (Su *et al.*, 2019).

Freely available bioinformatics tools have been developed for processing of NGS HIVDR testing data. Of these, the highly recommended include; PASeq (entirely web-based), HyDRA and MiCall which are accessible via webservers or command-line utilities. A study comparing the performance of NGS HIVDR analysis tools showed that HyDRA and MiCall have similar performance though HyDRA is more time efficient (Lee *et al.*, 2020). After data processing, drug resistance mutations are identified in reference to the Stanford University HIV drug resistance database (HIVdb) algorithm (Ji *et al.*, 2020). Due to the scarcity of bioinformatics expertise to locally analyze NGS-based HIVDR testing data in resource-limited settings, researchers in such settings mainly depend on the web-based tools (Ji *et al.*, 2018).

However, uploading NGS data onto webservers is associated with some challenges; (i) it is not reliable in settings without sustainable internet connectivity and (ii) once the data are uploaded, the data generator loses control of the data security which is critical for privacy of the patients.

Here, we present a portable and scalable all-in-one analysis pipeline that depends on open-source command-line utilities, and a local implementation of the HIVdb algorithm, which should be updated regularly to the latest release of the algorithm. An all-in-one pipeline that can fully run locally is important as it is reliable where instability of network connectivity is rife which is a common occurrence in resource-limited settings, and it ensures full control over data provenance in the context of varying data sharing laws some of which restrict data transfers across networks.

## 2 Methods

**Fig. 1.**
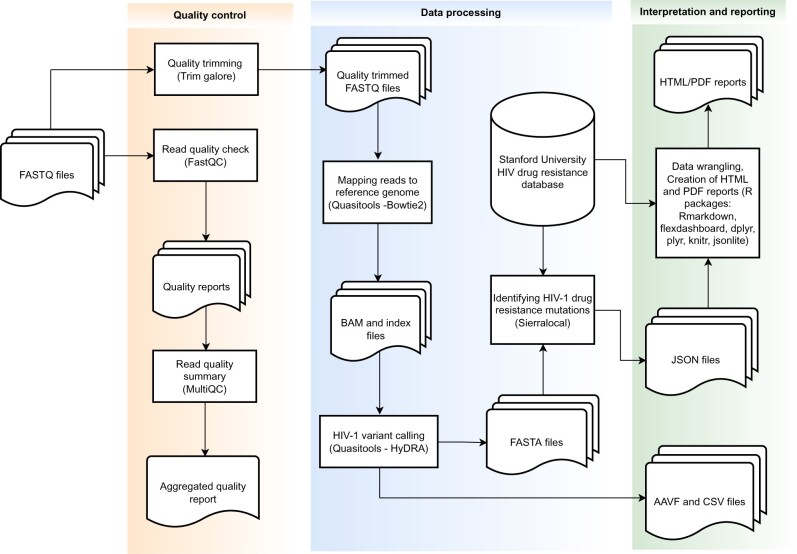
Workflow of QuasiFlow pipeline and example drug resistance report. QuasiFlow takes raw sequence reads in FASTQ format as input, performs quality control, mapping of reads to a reference genome, variant calling, querying the database for detection of HIV-1 drug resistance mutations and ultimately generates a user-friendly report in PDF/HTML format. AAVF, amino acid variant format; CSV, comma separated values

**Fig. 2.**
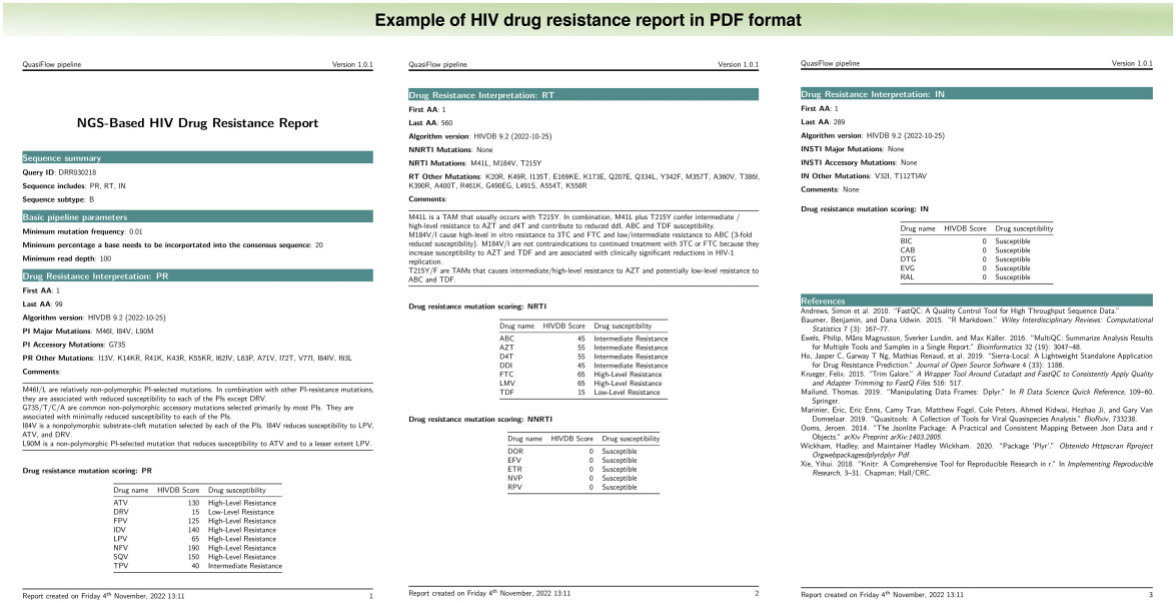
HIV drug resistance report. It consists of a sequence summary section which includes the sequence name, genome regions and the HIV-1 subtype; basic pipeline parameters including the minimum mutation frequency, minimum percentage and minimum read depth; drug resistance interpretation for protease inhibitors (PIs), non-nucleoside reverse transcriptase inhibitors (NNRTIs), nucleoside reverse transcriptase inhibitors (NRTIs) and integrase strand transfer inhibitors (INIs); references list for the tools used in the pipeline

QuasiFlow consists of a combination of bioinformatics tools as shown in the workflow (Fig. 1). The pipeline takes paired-end short reads from Illumina platforms as input. The quality of the reads is checked using FastQC (https://www.bioinformatics.babraham.ac.uk/projects/fastqc/) which generates a report for each of the input files. FastQC results are aggregated into a single report with MultiQC (Ewels *et al.*, 2016) for easy inspection. Adapter trimming of the reads is done using trim galore (Krueger, 2012). As part of Quasitools (https://phac-nml.github.io/quasitools/), Bowtie2 (Langmead and Salzberg, 2012) is used to align reads onto the HIV reference genome HXB2. A local implementation of HyDRA provided by quasitools is used for HIV-1 variant calling. Quasitools outputs filtered FASTQ files, amino acid variant call files, a mixed base consensus sequence in FASTA format and a drug resistance mutation report (consisting of identified drug resistance mutations and corresponding mutational frequencies) in comma separated values format. The consensus sequences are parsed onto sierralocal (Ho *et al.*, 2019) for scoring of identified drug-resistant mutations. Sierra-local generates a JSON object which is parsed to the R programming environment to generate a drug resistance report (Fig. 2). The tools are assembled into an automatic workflow using Nextflow (Di Tommaso *et al.*, 2017). QuasiFlow is distributed with docker containers for all third-party tools which enables its usage across multiple computing environments including Windows-, MacOS- and Linux-based systems. It is publicly available at https://github.com/AlfredUg/QuasiFlow and a corresponding vignette is available as Supplementary material.

## 3 Results

### 3.1 Testing and validation

The pipeline was tested with an updated version of the resistance genotyping algorithm (HIVdb 9.2). We used a publicly available dataset obtained from the National Center of Biotechnology Information (NCBI) Sequence Read Archive, Bio project accession PRJDB3502. These data comprised paired-end reads derived from 100 samples generated as part of the study that analyzed quasispecies of HIV-1 near full-length sequences (Ode *et al.*, 2015). The prevalence of at least one drug resistance mutation was 85% with a median number of drug resistance mutations of 3 and an interquartile range of 2–13 at mutation detection threshold (MDT) of 10%. We benchmarked QuasiFlow with two web-based systems that take FASTQ files as input including HyDRA web (https://hydra.canada.ca/analyses/) HIVdb-NGS (https://hivdb.stanford.edu/hivdb/by-sequences/) and one system that accepts consensus sequences in FASTA format as input, the classical Stanford University HIVdb program (https://hivdb.stanford.edu/hivdb/by-sequences/). For each sample, we obtained total drug resistance scores for antiretroviral drugs of the following drug classes; protease inhibitors (PIs), non-nucleoside reverse transcriptase inhibitors (NNRTIs), nucleoside reverse transcriptase inhibitors (NRTIs) and integrase strand transfer inhibitors (INIs).

Concordance between two systems was measured as the percentage of identical drug scores reported by both systems. Comparisons were made at four MDT, i.e. at 2%, 5%, 10% and 20% (standard Sanger sequencing threshold) considering a minimum read length of 100, average read quality of 30, minimum read depth of 100 and minimum variant quality of 30. Notably, QuasiFlow was 100% concordant with the classical Stanford University HIVdb program at MDT of 20%, 10% and 5%, reducing to 99.60% at MDT of 2%. QuasiFlow was 99.12% concordant with HyDRA web at MDT of 20%, 98.68% at MDT of 10%, 98.56% at MDT of 5% and 97.72% at MDT of 2%. Similarly, the concordance between QuasiFlow and HIVdb-NGS varied from 98.04% at MDT of 20%, 97.48% at MDT of 10%, 97.00% at MDT of 5% and 96.08% at MDT of 2% (Table 1). The observed discrepancies could be attributed to differences in sequence quality control strategies employed by the different systems as previously indicated by Lee *et al.* (2020) in a study that compared performance of NGS pipelines for HIV drug resistance.

**Table 1.** Concordance between QuasiFlow and web-based systems

Mutation detection threshold (%)	QuasiFlow and HIVdb web (%)	QuasiFlow and HIVdb-NGS (%)	QuasiFlow and HyDRA web (%)
20	100.00	98.04	99.12
10	100.00	97.48	98.68
5	100.00	97.00	98.56
2	99.60	96.08	97.72

### 3.2 Runtime analysis

Analysis was performed on a 64-bit workstation with 16 GB RAM and an Intel Core i7, 2.3 GHz processor. For near full-length HIV-1 genomic data (NFL dataset), it required 59.17 s for QuasiFlow to generate a drug resistance report for a pair of FASTQ files with an average of 316 250 reads per FASTQ file. For paired-end, data derived from the *pol* gene (*pol* gene dataset, Bioproject accession PRJNA559799) with an average of 50 686 reads per FASTQ file, it took an average of 21 s to generate a drug resistance report, which is about a third of the time required for a single sample from the NFL dataset.

In comparison with web-based systems, the average processing times for a pair of FASTQ files from the NFL dataset were 02 min 45 s and 01 min 44 s for HIVdb-NGS and HyDRA web, respectively. For the *pol* gene dataset, the processing times for a pair of FASTQ files were 01 min 2 s and 23 s for HIVdb-NGS and HyDRA web, respectively (Table 2).

**Table 2.** Performance comparison of QuasiFlow and web-based systems

Dataset	Time^a^	QuasiFlow	HyDRA web	HIVdb-NGS
*pol* dataset	Uploading	—	14 s	33 s
	Processing	21 s	09 s	29 s
NFL dataset	Uploading	—	∼01 min	∼02 min
	Processing	∼01 min	44 s	45 s

a Average time for uploading/processing a single sample with paired-end read data.

NFL, near full length.

## 4 Conclusion

We developed QuasiFlow, a portable and scalable pipeline for the reproducible analysis of NGS-based HIVDR testing data. QuasiFlow provides a single platform that can run fully locally/offline (with regular updates of the database to latest release of the algorithm) to expeditiously generate user-friendly HIVDR reports from raw NGS data. We hope this tool will improve reporting times of NGS-based HIVDR testing results, especially for researchers with unreliable internet connectivity (Jjingo *et al.*, 2022) and in cases where data transfer to remote servers is restricted.

## Supplementary Material

vbac089_Supplementary_Data

## Data Availability

The data used in this article were downloaded from the Sequence Read Archive (SRA), Bioproject accessions PRJDB3502 and PRJNA559799.
