# Supplementary material for: QuasiFlow: a Nextflow pipeline for analysis of NGS-based HIV-1 drug resistance data
Source: Bioinform Adv. 2022 Nov 28;2(1):vbac089. doi: 10.1093/bioadv/vbac089 (PMC9722223; doi:10.1093/bioadv/vbac089)
Supplement: vbac089_Supplementary_Data [file vbac089_supplementary_data.zip › Quasiflow-vignette.pdf]

QuasiFlow: A Nextflow Pipeline for Analysis of NGS-based HIV-1 Drug Resistance Data

Alfred Ssekagiri, Daudi Jjingo, Ibra Lujumba, Nicholas Bbosa, Daniel L. Bugembe, David P. Kateete, I. King Jordan, Pontiano Kaleebu, Deogratius Ssemwanga.

2022-08-15

Introduction

QuasiFlow is a nextflow pipeline for reproducible analysis of NGS-based HIVDR testing data across different computing environments. The pipeline takes raw sequence reads in FASTQ format as input, performs quality control, mapping of reads to a reference genome, variant calling, querying the database for detection of HIV drug resistance mutations, and ultimately generates a user-friendly report in PDF and HTML format. QuasiFlow is publicly available at https://github.com/AlfredUg/QuasiFlow.

Installation

QuasiFlow requires **nextflow** (version 21.04.3 or higher) and any of **conda**/**singularity**/**docker**. In this walk through, we shall demonstrate the use of `conda` which is more readily available to most users.

The first option is to install the pipeline using nextflow, it will be installed in the `$HOME` directory under the `.nextflow` sub-directory. Confirm that installation was successful by printing out the help message.

```
nextflow pull AlfredUg/QuasiFlow
nextflow run ~/.nextflow/assets/AlfredUg/QuasiFlow --help
```

Alternatively, simply clone the pipeline repository into a desired directory. Similarly, confirm that installation was successful by printing out the help message.

```
git clone https://github.com/AlfredUg/QuasiFlow.git
nextflow run QuasiFlow --help
```

Usage

The pipeline takes as input paired-end illumina data in `FASTQ` format. Let's download some test data from the European Nucleotide Archive (ENA) using `wget` command and decompress it using the `gunzip` command. This is a set of paired-end HIV-1 NGS data from a single sample which is part of bioProject PRJDB3502.

```
wget ftp://ftp.sra.ebi.ac.uk/vol1/fastq/DRR030/DRR030218/DRR030218_1.fastq.gz
wget ftp://ftp.sra.ebi.ac.uk/vol1/fastq/DRR030/DRR030218/DRR030218_2.fastq.gz
```

```
gunzip DRR030218*.gz
```

Run QuasiFlow on a test dataset with default parameters under the `conda` profile. This option does not require prior installation since it automatically pulls the pipeline from `main` branch of the pipeline repository on github. In addition, it installs all the dependancies in a `conda` environment. If you already installed the pipeline using the procedure above, see next options.

```
nextflow run AlfredUg/QuasiFlow -r main --reads "$PWD/*_{1,2}.fastq" -profile conda
```

Results will automatically be saved in the **results** sub-directory of the working directory. See the **pipeline outputs** below section for details.

If you pulled/installed the pipeline using nextflow, simply point to the installation path as follows;

```
nextflow run ~/.nextflow/assets/AlfredUg/QuasiFlow --reads "$PWD/*_{1,2}.fastq" -profile conda
```

Similarly, if you already cloned the pipeline repository, simply point to the installation path as follows;

```
nextflow run path/to/QuasiFlow --reads "$PWD/*_{1,2}.fastq" -profile conda
```

Profiles

Quasiflow can be run under different computing environments, simply choose an appropriate profile via the `-profile` argument. Could take any of the following `-profile conda, singularity, docker`. Note that, you need to `singularity` and `docker` should be installed prior to using their respective profiles.

```
nextflow run path/to/QuasiFlow --reads "$PWD/*_{1,2}.fastq" -profile singularity
```

Custom profiles can be added to the `conf` directory using any of the available profiles as a template.

Pipeline Outputs

Quality control

- `raw_reads_multiqc_report.html`: Aggregated quality control data and visualisations - one file for entire dataset

Variants and drug resistance outputs

- `consensus*.fasta`: `FASTA` files of consensus sequences - one per sample
- `consensus*.json`: `JSON` files of detailed HIV drug resistance analysis - one per sample
- `dr_report*.csv`: `CSV` files of drug resistance mutations at different mutational frequencies - one per sample
- `filtered*.fastq`: `FASTQ` files of drug resistance mutations at different mutational frequencies - one per sample
- `mutation_report*.aavf`: `AAVF` files of amino acid variant calls - one per sample
- `hivdr*.html`: `HTML` Final drug resistance report - one per sample

Pipeline information output

- `QuasiFlow_DAG.html`: Graphical representation of the pipeline's processes/operators and channels between them.
- `QuasiFlow_report.html`: Overall start and completion time, CPU and memory usage.
- `QuasiFlow_timeline.html`: Timeline for all the processes executed in the pipeline.

Note: Nextflow throws the following warning on MacOS, `WARN: Task runtime metrics are not reported when using macOS without a container engine`.

Parameters

HyDRA parameters

Mandatory parameters

- `--reads`: Path to input data (must be surrounded with quotes)

Optional parameters

- `--reporting_threshold`: Minimum mutation frequency percent to report.

- `--consensus_pct`: Minimum percentage a base needs to be incorporated into the consensus sequence.

- `--min_read_qual`: Minimum quality for a position in a read to be masked.
- `length_cutoff`: Reads which fall short of the specified length will be filtered out.
- `score_cutoff`: Reads that have a median or mean quality score (depending on the score type specified) less than the score cutoff value will be filtered out.
- `--min_variant_qual`: Minimum quality for variant to be considered later on in the pipeline.
- `--min_dp`: Minimum required read depth for variant to be considered later on in the pipeline.
- `--min_ac`: The minimum required allele count for variant to be considered later on in the pipeline
- `--min_freq`: The minimum required frequency for mutation to be considered in drug resistance report.

Sierralocal parameters

Optional parameters

- `--xml`: Path to HIVdb ASI2 XML.
- `--apobec-tsv`: Path to tab-delimited (tsv) HIVdb APOBEC DRM file.
- `--comments-tsv`: Path to tab-delimited (tsv) HIVdb comments file.

Output parameters

Optional parameters

- `--outdir`: Path to directory where results will be saved

Dependancies.

Below is the list of tools that are used in the QuasiFlow pipeline. These tools are readliy available and may be installed using `conda` via `bioconda` channel.

- fastQC
- MultiQC
- Trim-galore
- Quasitools
- Sierra-local
- R packages (Jsonlite, plyr, dplyr, flexdashboard, rmarkdown, knitr, tinytex), all available in CRAN

Troubleshooting

Kindly report any issues at https://github.com/AlfredUg/QuasiFlow/issues.

License

QuasiFlow is licensed under GNU GPL v3.

Citation

This work is currently under peer review. A formal citation will be availed in due course.
